# Sedentary Habits

**Published:** 1874-03

**Authors:** 


### SEDENTARY HABITS.

A MAN may be healthy without being strong; but all health tends, more or less, towards strength, and all disease is weakness. Now, any one may see in Nature that things grow big simply by growing; this growth is a constant and habitual exercise of vital or vegetative force, and whatever checks or diminishes the action of this force—say harsh winds or frost—will stop the growth and stunt the production. Let the student, therefore, bear in mind that sitting on a chair, leaning over a desk, poring over a book, cannot possibly be the way to make his body grow. The blood can be made to flow, and the muscles to play freely only by exercise and if that exercise is not taken, Nature will not be mocked. Every young student ought to make a sacred resolution to move about in the open air at least two hours every day. If he does not do this, cold feet, the clogging of the wheel of the internal parts of the fleshy frame, and various shades of stomachic and cerebral discomfort, will not fail in due season to inform him that he has been sinning against Nature, and, if he does not mend his course as a bad boy, he will certainly be flogged, for Nature is never like some soft hearted human masters—over merciful in her treatment. But why should a student indulge so much in the lazy and unhealthy habit of sitting? A man may think as well standing as sitting, often not a little better; and as for reading in these days, when the most weighty books may be had cheaply in the lightest form, there is no necessity why a person should be bending his back and doubling his chest merely because he happens to have a book in his hand. A man will read a play or poem far more naturally and effectively while walking up and down the room than when sitting sleepily in a chair. Sitting, in fact, is a slovenly habit, and ought not to be indulged. But when a man does sit, or must sit, let him at all events sit erect, with his back to the light, and a full free projection of the breast. Also, when studying languages, or reading fine passages of poetry, let him read as much as possible aloud; a practice recommended by Clemens, of Alexandria, and which will have the double good effect of strengthening that most important vital element, the lungs, and training the ear to the perception of vocal distinction, so stupidly neglected in many of our public schools. There is, in fact, no necessary connection, in most cases, between the knowledge which a student is anxious to acquire and the sedentary habits which students are apt to cultivate.—"*On Self-Culture*," *by Professor Blackie.*

